# Evidence-Based Aerobic Exercise Training in Metabolic-Associated Fatty Liver Disease: Systematic Review with Meta-Analysis

**DOI:** 10.3390/jcm10081659

**Published:** 2021-04-13

**Authors:** Joanna Słomko, Marta Zalewska, Wojciech Niemiro, Sławomir Kujawski, Maciej Słupski, Beata Januszko-Giergielewicz, Monika Zawadka-Kunikowska, Julia Newton, Lynette Hodges, Jacek Kubica, Paweł Zalewski

**Affiliations:** 1 Department of Hygiene, Epidemiology, Ergonomy and Postgraduate Education, Ludwik Rydygier Collegium Medicum in Bydgoszcz Nicolaus Copernicus University, 85-094 Bydgoszcz, Poland; skujawski@cm.umk.pl (S.K.); m.zkunikowska@cm.umk.pl (M.Z.-K.); p.zalewski@cm.umk.pl (P.Z.); 2 Department of Prevention of Environmental Hazards and Allergology, Medical University of Warsaw, 02-091 Warsaw, Poland; mzalewska@wum.edu.pl; 3 Faculty of Mathematics, Informatics and Mechanics University of Warsaw, 02-097 Warsaw, Poland; wniemiro@gmail.com; 4 Faculty of Mathematics and Computer Science Nicolaus Copernicus University, 87-100 Torun, Poland; 5 Department of Hepatobiliary and General Surgery, Faculty of Medicine Collegium Medicum in Bydgoszcz, Nicolaus Copernicus University, 85-094 Bydgoszcz, Poland; maciej.slupski@cm.umk.pl (M.S.); b.giergielewicz@cm.umk.pl (B.J.-G.); 6 Population Health Sciences Institute, The Medical School, Newcastle University, Newcastle-upon-Tyne NE2 4AX, UK; julia.newton@ahsn-nenc.org.uk; 7 School of Sport, Exercise and Nutrition, Massey University, Palmerston North 4442, New Zealand; l.d.hodges@massey.ac.nz; 8 Department of Cardiology and Internal Medicine, Nicolaus Copernicus University Collegium Medicum, 85-094 Bydgoszcz, Poland; jkubica@cm.umk.pl

**Keywords:** fatty liver, metabolic fatty liver, aerobic activity, exercise, treatment

## Abstract

Background: This meta-analysis evaluates the overall effect of the non-pharmacological intervention, aerobic exercise, upon serum liver enzymes levels, glucose metabolism and anthropometric measures amongst patients with metabolic associated fatty liver disease (MAFLD). It also examines whether the effects on these outcomes are moderated by the aerobic training protocol when considered according to the American College of Sports Medicine (ACSM) recommended FITT (frequency, intensity, time, type) principles. Approach and Results: Fifteen randomized control trials were included in the meta-analysis. Compared with usual care, continuous and interval training showed significant efficacy in alanine aminotransferase (ALT) level improvement (MD = −2.4, 95% CI: −4.34 to −0.46 *p* = 0.015, I^2^ = 9.1%). Interventions based on all types of aerobic exercise protocols showed significant improvement of intrahepatic triglycerides (MD = −4.0557, 95% CI: −5.3711 to −2.7403, *p* < 0.0001, I^2^ = 0%) and BMI (MD = −0.9774, 95% CI: −1.4086 to −0.5462, *p* < 0.0001, I^2^ = 0). Meta-regression analysis demonstrated a significant correlation between total intervention time and ALT level (for all aerobic protocols: 6.0056, se = 2.6896, z = 2.2329, *p* = 0.02; as well as for continuous and interval aerobic protocols: 5.5069, se = 2.7315, z = 2.016, *p* = 0.04). Conclusions: All types of aerobic exercise protocols are effective at improving intrahepatic triglycerides and lead to a reduction in body mass index. In addition, continuous and interval aerobic exercise may be more effective at improving ALT ≤12 weeks intervention time benefits the management of MAFLD.

## 1. Introduction

Metabolic associated fatty liver disease (MAFLD), formerly named non-alcoholic fatty liver disease (NAFLD), represents the hepatic manifestation of a multisystem disorder and is a leading cause of chronic liver disease worldwide [1]. An international expert consensus statement from 2020 proposed diagnostic criteria for MAFLD, which are based on histological, imaging or blood biomarker evidence of hepatic steatosis, with at least one of the following additional criteria: overweight or obesity, type 2 diabetes mellitus or metabolic dysregulation (at least two metabolic risk abnormalities): waist circumference ≥102/88 cm in Caucasian men and women or ≥90/80 cm in Asian men and women, blood pressure ≥130/85 mmHg or specific drug treatment, plasma HDL-cholesterol <40 mg/dl for men and <50 mg/dl for women or specific drug treatment, prediabetes, homeostasis model assessment of insulin resistance score ≥2.5 and plasma high-sensitivity C-reactive protein level >2 mg/L [2].

The global prevalence of NAFLD is reported to be from 13.48% in Africa, 23.71% in Europe, 24.13% in North America, 27.37% in Asia to 31.79% in the Middle East and represents a major health problem and an important economic burden with currently a lack of effective pharmacological therapy [3,4]. The annual direct medical costs associated with NAFLD were estimated to be €35 billion in Europe and €89 billion in the USA [5]. Early diagnosis, prevention and management of disease-related risk factors as well as lifestyle modifications have been proposed as cost-effective strategies for MAFLD treatment [6,7]. The 2016 European Association for the Study of the Liver guidelines recommends lifestyle interventions based on combined dietary restriction and a progressive increase in aerobic exercise or resistance training [8]. However, recent evidence suggests that increased physical activity participation can lead to favorable health benefits for patients with NAFLD and recommends an independent role for physical activity alone as an intervention for patients with NAFLD [9,10]. In addition, aerobic (such as walking or cycling) and resistance exercise programs reduce hepatic steatosis and improve several health outcomes in patients with NAFLD, leading to reduced cardiovascular risk, the main cause of mortality in this population [11].

Many studies performed on animals models have indicated that aerobic exercise improves hepatic lipid metabolism in NAFLD by affecting lipid synthesis, reducing mitochondrial depended apoptosis, improving oxidative metabolism and decreasing steatosis and hepatic inflammation [12,13]. Moreover, previous meta-analyses highlight a range of beneficial effects of physical exercise on liver fat [14,15,16,17,18,19].

Although exercise is the first-line therapy for patients with metabolic associated fatty liver disease, the optimal exercise program with required frequency, intensity and duration remains unclear, and the mechanisms by which exercise affects the liver remain, at least in part, unknown [20,21]. The American College of Sports Medicine (ACSM) defines a standard physical exercise program prescription to include the FITT (frequency, intensity, time, type) principles [22]. More research is needed to determine what dose of physical activity assessed by the FITT (frequency, intensity, time, type) principles provide the greatest health benefits in MAFLD.

The aims of the current meta-analysis are: (1) to determine the overall effect of aerobic exercise on changes in serum levels of liver enzymes, glucose metabolism and anthropometric changes in patients with MAFLD; (2) to examine whether the effects on these outcomes are moderated by the aerobic training protocol according to ACSM criteria, intensity, progression, frequency, duration and length of treatment; (3) to explore the relationship between the dose of physical activity and the effectiveness of the intervention.

## 2. Materials and Methods

The protocol for this systematic review and meta-analysis was based on the Preferred Reporting Items of Systematic Reviews and Meta-Analysis (PRISMA) statement [23]. The design of the present work was fully specified in advance. It was registered in the PROSPERO (International Prospective Register of Systematic Reviews, CRD42020211873).

### 2.1. Eligibility Criteria 

#### 2.1.1. Types of Participants 

Studies were included if they were conducted in adult patients (aged >18) of any gender or nationality with biopsy-proven or imaging-proven fatty liver disease with obesity or overweight (BMI >25 kg/m^2^ in white and >23 kg/m^2^ in Asian individuals), type 2 diabetes mellitus or evidence of metabolic dysregulation.

#### 2.1.2. Types of Interventions 

Studies included an arm of an aerobic exercise component (continuous, interval or combination) targeting MAFLD management. The interventions were considered aerobic as defined by ACSM.

#### 2.1.3. Types of Comparisons 

Studies included a control condition, consisting of usual care or another type of intervention.

#### 2.1.4. Types of Outcomes 

Studies presented statistical data allowing at least one of the following outcomes—changes in serum levels of liver enzymes: alanine aminotransferase (ALT), aspartate aminotransferase (AST), gamma-glutamyl transpeptidase (GGT); intrahepatic triglycerides (IHTG), glucose metabolism: homeostatic model assessment of insulin resistance (HOMA-IR, HOMA2-IR) other outcome measures included anthropometric changes (BMI, body mass index), measured at baseline (pre-treatment) and at post-treatment.

#### 2.1.5. Types of Studies 

Studies were included if they were randomized controlled trials (RCTs) published in peer review journals in English. There were no restrictions with respect to the length of the intervention and follow-up measurement point(s).

The exclusion criteria were as follows: non-RCT, case reports, reviews, non-human, trials with secondary hepatic steatosis enrollment, non-information about training program (intensity, frequency and duration). The PICOS criteria for inclusion and exclusion of studies are shown in Table 1 [24].

### 2.2. Search Strategy and Study Selection 

Initially, electronic databases (PubMed, Scopus, Cochrane Library, ClinicalKey) were searched for relevant articles published between 2005 and 22 December, 2020. The combination of search terms was: (“NAFLD” OR “non-alcoholic fatty liver” OR “fatty liver”) AND (“aerobic” OR “aerobic exercise” OR “aerobic exercise training”).

Two authors (SK, MZK) read the titles and abstracts retrieved. If the studies appeared to meet the inclusion criteria, full texts were obtained and reviewed by the first author (JS). Next, reference lists from previous review articles and included studies were hand-searched to find additional studies. The senior author (PZ), using an international expert consensus statement from 2020, checked diagnostic criteria for metabolic associated fatty liver disease in the included studies and approved the final selection of studies.

### 2.3. Quality and Risk of Bias Assessment 

Study quality was evaluated using Cochrane Risk of Bias Tools which cover six domains of bias: selection bias (random sequence generation, allocation concealment), performance bias (blinding of participants and personnel), detection bias (blinding of outcome assessment), attrition bias (incomplete outcome data), reporting bias (selective reporting) and other bias [25]. Funnel plots have been used to provide a visual assessment of the association between treatment estimate and study size. Publication bias was considered significant when *p*-value was less than 0.05 in either Begg’s test [26] (Supplemetary Figure S1).

### 2.4. Statistical Analysis

Analyses were conducted using meta-packages of R. [26,27,28,29]. Both, random and fixed effects models were used; a random-effect model was used to estimate the pooled effect when I^2^ values were ≥50%. The effect size was calculated as the mean difference (MD) changes from baseline along with 95% confidence intervals (CI). A statistically significant *p*-value was based on <0.05. Data of each indicator was pooled and shown as a forest plot. Heterogeneity was tested using the Cochran’s Q test and measured inconsistency by I^2^ (I^2^ values >50% were defined as high heterogeneity, between 25 and 50% as moderate heterogeneity, and <25% as low heterogeneity). Meta-regression was performed to explore the possible correlation between the dose of physical activity (intervention time: more or less than 12 weeks, intensity: moderate or vigorous, volume: more or less than 180 min/week) and the effectiveness of the intervention [27,28,29].

## 3. Results

### 3.1. Study Selection

**Figure 1:**
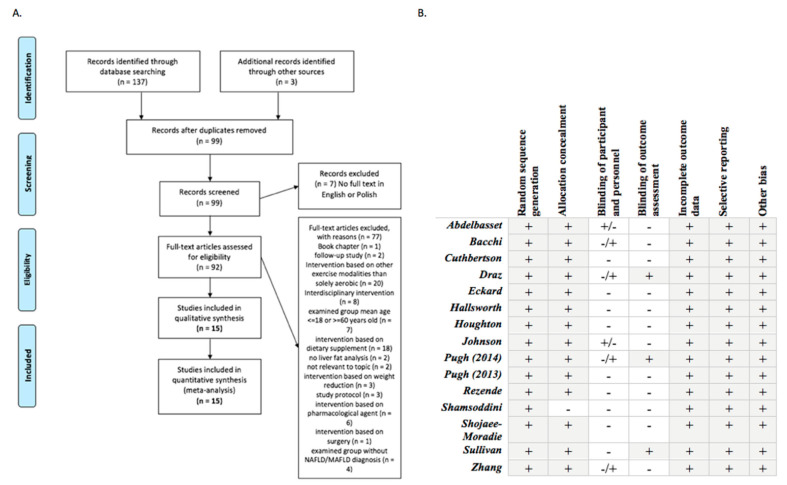
Flowchart of studies and critical appraisal. (**A**). Flow chart of the inclusion/exclusion process, according to the Preferred Reporting Items for Systematic Reviews and Meta-Analyses (PRISMA) statement [23]. (**B**). Quality of the trials and Cochrane risk bias [24].

In total, 140 studies were initially retrieved (Figure 1); of those, 15 RCT’s met the inclusion criteria. These reports covered 7 countries: 6 from the UK, two from the USA and one from each of the following countries: China, Australia, Italy, Iran, Egypt, Saudi Arabia and Brasil. Table 1 shows the characteristics of the trials included in the meta-analysis.

### 3.2. Participant Characteristics

740 MAFLD patients (383 treatment group: mean age of 51 years, mean BMI 32.2 kg/m^2^; 357 control group) were included in the meta-analysis. Table 2 shows the patient characteristics. In seven trials, MAFLD were diagnosed by ^1^H MRS-proven fatty liver disease who were overweight or obese [30,31,32,33,34,35,36]. In three other trials, MAFLD was diagnosed via ultrasound with overweight or obesity [37,38,39]. One trial included those with ultrasound-proven MAFLD with diabetes and obesity [40] and three were biopsy-proven with obesity [41,42,43].

### 3.3. Intervention; Comparison to ACSM Guidelines Characteristics

Table 3 shows the characteristics of the exercise protocols of the trials included in the meta-analysis. Intervention time ranged from 4 to 24 weeks, and exercise volume ranged from 30 min × 3 days per week to 60 min × 7 days per week. Twelve studies evaluated the efficacy of aerobic training versus standard care [30,31,32,33,35,36,37,39,40,41,42,43], three studies evaluated the efficacy of aerobic training versus another type of intervention: stretching [34], resistance training [44] and electroacupuncture [38].

According to ACSM’s guidelines, studies were categorized by intensity: continuous very light training, continuous light training, continuous moderate training, continuous vigorous and interval training; volume: low to moderate, moderate to high and gradual progression of exercise volume by adjusting exercise duration or intensity or duration, frequency, and intensity. For the prescription of aerobic exercise, in nine studies, exercise intensity was obtained from cardiopulmonary exercise test (CPET) [30,31,32,33,34,38,39,40,41]; in two studies HR_max_ was calculated using the formula HR_max_ = 220–age [36,37]; in two study intensity was estimated using Borg rating of perceived exertion (RPE) [35,43] and in one study HRR using Karvonen formula [44].

**Figure 2:**
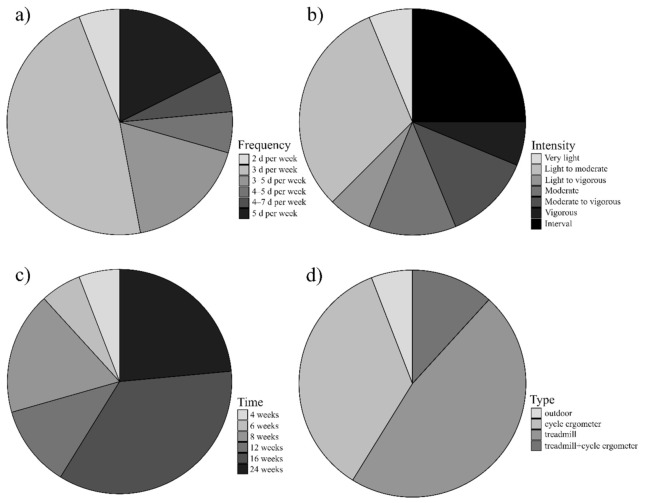
Details of included aerobic protocols according to FITT principle recommended by ACSM. (**a**) Frequency: eight protocols 3 d per week, three protocols 3–5 d per week, three protocols 5 d per week and one from each of the following: 4–7 d per week, 4–5 d per week, 2 d per week; (**b**) Intensity: five protocols light to moderate, 4 protocols interval training, two protocols moderate, two protocols moderate to vigorous, one from each of the following: light to vigorous, very light, vigorous; (**c**) Time: six protocols 16 weeks, four protocols 24 weeks, two protocols 12 weeks, two protocols 8 weeks, one from each of the following: 6 and 4 weeks; (**d**) Type: eight protocols treadmill, six protocols cycle ergometer, two protocols treadmill with cycle ergometer and one outdoor training.

In addition, eight studies implemented continuous moderate training [30,31,32,33,34,37,40,44], four of them were more than 180 min/week with 16 weeks intervention time and with gradual progression of exercise volume by adjusting exercise duration, frequency, and intensity; four studies reported an exercise volume program of 120–180 min/weeks with intervention period time 4–16 weeks, Table 3, Figure 2 (two of them reported gradual progression of exercise volume by adjusting exercise intensity). One study implemented very light continuous training, 150 min per week with an intervention time 24 weeks [36]. Three studies implemented interval training from six to twelve weeks [35,38,40]. Three studies implemented combination training [39,42,43], two of them continuous aerobic training with resistance exercise and one interval training with resistance exercise, 80–420 min per week, from twelve to twenty-four weeks (Table 3).

Intervention in four of the seventeen protocols met all domains of the FITT criteria recommended by ACSM [33,34,35,36]. Another six met criteria progressively over time, whereby the intensity increased as the intervention advanced forward [30,31,32,38,39,40]. Therefore, in ten protocols, all FITT domains were met by the end of treatment. Two protocols met three of the ACSM FITT criteria (Table 3).

### 3.4. Effect of Aerobic Exercise on Changes in Serum Levels of Liver, Intrahepatic Triglycerides, Glucose Metabolism and Body Mass Index 

All studies assessed changes in serum levels of liver enzymes using ALT [30,31,32,33,34,35,36,37,38,39,40,41,42,43,44], twelve RCT’s used AST [30,31,32,35,36,37,38,39,41,42,43,44], eight RCT’s included GGT [30,31,32,35,39,41,43,44].

Four RCT’s measured IHTG assessed by ultrasound or ^1^H MRS or improvement in liver histology estimated by the NAFLD activity score (NAS) [34,36,40,43].

Glucose metabolism: six RCT’s using HOMAR-IR [31,34,37,40,41,43], five RCT’s HOMA 2-IR [30,31,34,35,40]; twelve RCT’s assessed anthropometric changes using body mass index (BMI) [30,31,32,33,34,35,37,39,40,41,43,44].

**Figure 3:**
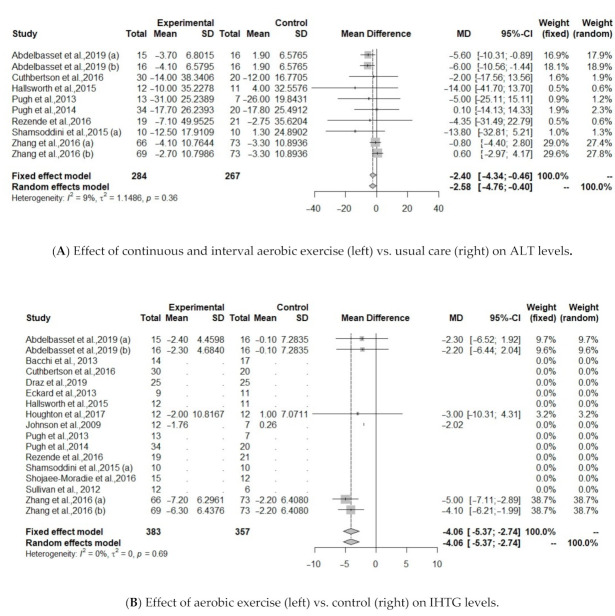

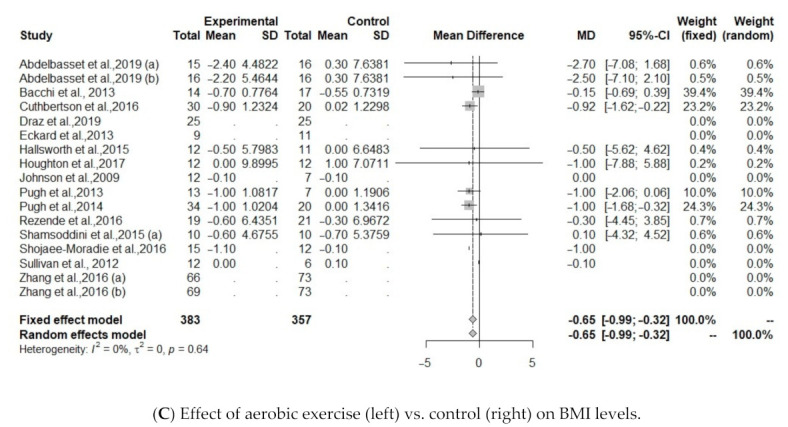
(**A**). Effect of continuous and interval aerobic exercise (left) vs. usual care (right) on ALT levels in patients with MAFLD; ALT, alanine aminotransferase. (**B**). Effect of exercise (left) vs. control (right) on IHTG levels; IHTG, intrahepatic triglycerides. (**C**). Effect of exercise (left) vs. control (right) on BMI levels; BMI, body mass index.

Regarding serum liver enzymes, intervention based on all type of aerobic exercise protocols (continuous, interval, combination) did not improve ALT, AST, GGT levels (Table 4). Heterogeneity of the effect measures regarding ALT (I^2^ = 39.9, *p* = 0.06) and AST (I^2^ = 35.7, *p* = 0.10) was moderate; for GGT (I^2^ = 0%, *p* = 0.89) was low. When continuous and interval aerobic exercise was compared to usual care, analysis from 10 protocols showed improvements on ALT (MD = −2.4, 95% CI: −4.34 to −0.46 *p* = 0.015, I^2^ = 9.1%), Table 4, Figure 3A.

Five protocols investigated IHTG between treatment and control group [34,36,40,43]. Results showed that IHTG was significantly reduced after aerobic exercise (MD = −4.0557, 95% CI: −5.3711 to −2.7403, *p* <0.0001), including 190 individuals (Figure 3B).

The effect of aerobic exercise on BMI was studied in 10 of the identified protocols [30,31,32,34,35,37,40,41,43,44]. Results showed a significant association between aerobic exercise group and controls (MD = −0.9774, 95% CI: −1.4086 to −0.5462, *p* <0.0001). Heterogeneity was low regarding IHTG (I^2^ = 0%, *p* = 0.69) and BMI (I^2^ = 0%, *p* = 0.99), Figure 3C.

There were no significant changes in HOMA-IR and HOMA2-IR. Heterogeneity was low for HOMA-IR (I^2^ = 12.7%, *p* = 0.22, but high for HOMA2-IR (I^2^ = 71.7%, *p* = 0.03).

### 3.5. Meta-Regression Analysis Result

Meta-regression analysis demonstrated a significant correlation between total intervention time and ALT difference such that the shorter intervention time (≤12 weeks), the more effective the aerobic exercise intervention at lowering ALT (for all aerobic protocols: 6.0056, se = 2.6896, z = 2.2329, *p* = 0.02; for continuous and interval aerobic protocols: 5.5069, se = 2.7315, z = 2.016, *p* = 0.04). Other factors that we assessed did not significantly impact the magnitude of the AST (intervention time *p* = 0.34, intensity *p* = 0.53), GGT (intensity *p* = 0.84), IHTG (intervention time *p* = 0.32) and BMI (intervention time *p* = 0.43, intensity *p* = 0.38, volume *p* = 0.37); detailed information about meta-regression analysis result are available in Supplementary Table S1.

## 4. Discussion

Our meta-analysis summarizes the findings of fifteen RTC’s in MAFLD management and provides detailed evidence of the role of aerobic exercise in MAFLD patients diagnosed with criteria from an international expert consensus statement from 2020 [2].

Based on the current literature, aerobic exercise can offer important health benefits [45]. The World Health Organization (WHO) recommends that adults should perform at least 150–300 min of moderate-intensity or 75–150 min of vigorous exercise per week for optimum health [46]. ACSM and the Centers for Disease Control, the National Institutes of Health concluded that moderate-intensity aerobic activity is effective in reducing the overall risk of chronic disease [47]. Moreover, the American College of Sports Medicine and the American Diabetes Association recommends at least 150 min/week of moderate-intensity or 75 min/week of vigorous-intensity physical activity for all adults to reduce the risk of cardiovascular disease and type 2 diabetes as well as to improve cardiorespiratory fitness [48]. However, it remains unknown whether the guideline-recommended minimum levels of physical activity, are also sufficient to reduce the risk of MAFLD, and if there exists a dose–response relationship.

Our results show that 35% (4/17) of aerobic protocols met all of the ACSM guidelines for aerobic exercise prescription. The likely reason why the studied interventions did not strictly follow aerobic exercise guidelines is related to the perceived lack of adherence for patients with MAFLD to the guidelines. Results from other studies show that physical activity levels are lower in NAFLD patients than those without [49,50]. Krasnoff et al. found that more than 80% of NAFLD patients did not complete a physical activity program of 30 min moderate-intensity exercise 3 times per week [51]. Moreover, Hallsworth and Adams suggest that optimal FITT recommendations for MAFLD patients are unclear and clinical guidelines are not disease-specific. Therefore, an effective physical activity program for MAFLD should meet individual patient needs [11].

Our results suggest that all types of aerobic exercise: continuous, interval and combination, compared to usual care or another type of intervention, has a large effect on IHTG levels and BMI. Moreover, continuous and intermittent aerobic exercise compared to usual care improved liver enzyme levels, specifically, alanine aminotransferase (ALT). Importantly, meta-regression analysis has shown that a shorter intervention time (≤12 weeks) is more effective in ALT reduction. Other factors, i.e., frequency, intensity, volume and progression of the exercise protocol did not significantly impact upon the magnitude of the measured parameters. No difference between intensity levels, frequency or training volume was seen to reduce hepatic steatosis, suggesting different combinations of aerobic exercise may be equally beneficial. A number of studies provide evidence that aerobic exercise indeed reduces hepatic fat content at various intensity doses and frequencies [52,53,54,55]. Keating et al. investigated the effects of different doses of physical activity on NAFLD management and found that low to moderate-intensity aerobic training 90–135 min per week or 180–240 min per week is as effective in reducing hepatic and visceral fat as high-intensity aerobic training 90–135 min per week. [49] However, the mechanisms by which exercise reduces liver fat are still not fully understood [50].

The results of our study are consistent with a previous meta-analysis. Smart et al. showed that physical exercise programs, irrespective of dietary intervention, might be beneficial in terms of changes in intrahepatic fat, body mass, BMI < FFA, insulin liver enzymes, lipids and VO_2_ peak [16]. However, no significant reduction in liver enzymes was observed [16]. Interestingly, the authors conclude that exercise programs where total caloric expenditure is greater than 10,000 kcal, could be more effective in intrahepatic fat reduction than programs with lower caloric expenditure. In contrary to Smart et al., Katsagoni et al. noted significant effects of a physical exercise program upon liver enzyme reduction. Effects on AST and ALT seemed to be dependent on weight loss [19]. Continuous moderate-intensity aerobic exercise programs with higher volume (higher than 180 min per week) were more effective in intrahepatic triglycerides reduction than moderate-intensity aerobic exercise with volumes 120 to 180 min per week or HIIT [19]. Orci et al.’s meta-regression analysis indicated that the higher BMI before the intervention, the greater reduction of intrahepatic lipid content due to physical activity [14]. Moreover, interventions based on physical activity led to reductions of both ALT and AST. Physical activity significantly reduced intrahepatic liver content. In contrary to Katsagoni et al., the authors proposed that aerobic training programs might be a more effective modality in intrahepatic liver content reduction than resistance exercise [14].

Our study has several strengths. First, this meta-analysis provides detailed evidence of the role of aerobic exercise in improving liver function outcomes in MAFLD patients. As seen in other studies, we tried to detect a significant correlation between the dose of physical activity (according to FITT principle recommended by ACSM) and the effectiveness of the intervention. Our results suggest that ≤12 weeks intervention time benefits the management of MAFLD. This finding indicates a causal relationship between the time of aerobic exercise and ALT reduction. Second, the trials included in the meta-analysis are characterized by a low risk of bias according to the Cochrane Risk of Bias assessment tool and the heterogeneity among the available studies was low.

The strengths of this meta-analysis should be weighed against some limitations. First, because of the unavailability of comprehensive data in most studies the outcomes in our study were all restricted to the serum levels of liver enzymes, glucose metabolism and body mass index changes. Second, the current number of published studies does not allow analysis of the role of all components of FITT principle on liver function. Meta-regression between changes in IHTG, GGT, HOMA2-IR, HOMA-IR and different types of exercise protocol was considered, but changes in these parameters were not reported in all studies. Third, many of the studies that were included have only a few patients and only studies published in English were included in the present meta-analysis.

## 5. Conclusions

The current meta-analysis, based on RCT’s, provides strong evidence that aerobic exercise improves serum levels of alanine aminotransferase (ALT), intrahepatic triglycerides (IHTG) and anthropometric changes (BMI; body mass index), measured at baseline and post-treatment in MAFLD patients diagnosed using criteria from an international expert consensus statement from 2020. Aerobic exercise for ≤12 weeks intervention time as part of lifestyle management improves MAFLD pathophysiology and is more effective in ALT reduction than aerobic exercise protocols with longer intervention time.

Considering that the optimal and detailed exercise prescription (i.e., intensity, frequency, volume) is still unclear. This means that the clinical guidelines and FITT recommendations are non-specific and future original studies focused on the dose-related effect of exercise, are warranted.

## Figures and Tables

**Table 1:** PICOS criteria for inclusion and exclusion of studies.

Parameter	Defined criteria for the current study
P (population)	Adult patients with MAFLD
I (intervention)	Aerobic exercise
C (comparison)	Usual care or another type of intervention
O (outcomes)	Primary: changes in serum levels of liver enzymes, intrahepatic triglycerides Secondary: glucose metabolism, anthropometric changes
S (study design)	Randomized clinical trials

**Table 2:** Details of included studies.

First Author, Year	No of pts		MAFLD Definition	Age Years	BMI kg/m^2^	Female/Male	Treatment/ Control	Endpoints
	TG	CG						
Abdelbasset et al., 2019	15	16	Ultrasound; Diabetes, Obesity	54.9	36.7	7/8	CAEx/UC	ALT, HOMA2-IR, HOMA-IR, BMI, IHTG
Abdelbasset et al., 2019	16	16	Ultrasound; Diabetes, Obesity	54.4	36.3	6/10	IEx/UC	ALT, HOMA2-IR, HOMA-IR, BMI, IHTG
Bacchi et al., 2013	14	17	Diabetes	55.6	30.5	56	CAEx/REx	ALT, AST, GGT, BMI
Cuthbertson et al., 2016	30	20	^1^H MRS (>5.3% IHCL); Obesity	50.0	30.7	7/23	CAEx/UC	ALT, AST, GGT, HOMA2-IR, BMI
Draz et al., 2019	25	25	Ultrasound; Obesity	30-55	37.8	25/0	IEx/EAc	ALT, AST
Eckard et al., 2013	9	11	Biopsy; Obesity	52	31.3	3/6	CAEx+REx/UC	ALT, AST
Hallsworth et al., 2015	12	11	^1^H MRS (>5% IHTG); Obesity	54.0	31	N/R	IEx/UC	ALT, AST, GGT, HOMA2-IR, BMI
Houghton et al., 2016	12	12	Biopsy; Obesity	54			IEx+REx/UC	ALT, AST, GGT, HOMA-IR, BMI, IHTG
Johnson et al., 2009	12	7	^1^H MRS; Obesity	49.1		N/R	CAEx/St	ALT, HOMA2-IR, HOMA-IR, BMI, IHTG
Pugh et al., 2014	34	20	^1^H MRS (IHTG >5.5%); Obesity	48	31	12/22	CAEx/UC	ALT, AST, GGT, HOMA2-IR, HOMA-IR, BMI
Pugh et al., 2013	13	7	^1^H MRS (IHTG >5.5%); Obesity	50	31	6/7	CAEx/UC	ALT, AST, GGT, BMI
Rezende et al., 2016	19	21	Biopsy; Obesity	56.2	34.1	19/0	CAEx/UC	ALT, AST, GGT, HOMA-IR, BMI
Shamsoddini et al., 2015	10	10	Ultrasound; Overweight	39.7	28.1	0/10	CAEx/UC	ALT, AST, HOMA-IR, BMI
Shojaee-Moradie et al., 2016	15	12	Ultrasound or liver biopsy; Obesity	52.4	31.6	0/15	CAEx+REx/UC	ALT, AST, GGT, BMI
Sullivan et al., 2012	12	6	^1^H MRS (IHTG >10%); Obesity	48.6	37.1	8/4	CAEx/UC	ALT, BMI
Zhang et al., 2016 (a)	66	73	Ultrasound, ^1^H MRS (>5% IHTG); Overweight	53.2	27.9	52/14	CAEx/UC	ALT, AST, IHTG
Zhang et al., 2016 (b)	69	73	Ultrasound, ^1^H MRS (>5% IHTG); Overweight	54.4	28.1	51/18	CAEx/UC	ALT, AST, IHTG

TG, treatment group; CG, control group; BMI, body mass index; CAEx, continuous aerobic exercise; Rex, resistance exercise; IEx, interval exercise; EAc, electroacupuncture; St, stretching; UC, usual care; ALT, alanine aminotransferase; AST, aspartate aminotransferase; GGT, gamma-glutamyl transpeptidase; IHTG, intrahepatic triglycerides; HOMA-IR, HOMA2-IR, homeostatic model assessment of insulin resistance; ^1^H MRS proton magnetic resonance spectroscopy.

**Table 3:** Details of aerobic exercise protocols (according to FITT criteria).

	Frequency	Intensity	Type	Time	Progression	Duration	Volume	ACSM Criteria Met * ?
Abdelbasset et al., 2019	3 d per week	60–70% HRmax	Continuous training, Three phases: warm-up, training and cool down; Cycle ergometer	8 weeks	None	40–50 min	120–150 min/week	No
Abdelbasset et al., 2019	3 d per week	50–85% VO2max	Interval training, Three phases: warm-up, training and cool down; Cycle ergometer	8 weeks	None	40 min	120 min/week	Progressively yes
Bacchi et al., 2013	3 d per week	60–65% HRR	Continuous training, Cycle, treadmill; CG: resistance training	16 weeks	None	60 min	180 min/week	No
Cuthbertson et al., 2016	3–5 d per week	3 weeks 30% HRR; 5 week at 60% HRR by week 12	Continuous training, Treadmill, cross-trainer, cycle ergometer, rower	16 weeks	Duration, frequency, intensity	30–45 min	90–225 min/week	Progressively yes
Draz et al., 2019	3 d per week	60–85% HRmax	Interval training, Three phases: warm-up, training and cool down; Cycle ergometer, CG: electroacupuncture	6 weeks	None	30 min	90 min/week	Progressively yes
Eckard et al., 2013	4–7 d per week	N/A	Combination training, Cycle, Treadmill, resistance training	24 weeks	Frequency, intensity, time	20–60 min	80–420 min/week	No
Hallsworth et al., 2015	3 d per week	6–20 point Borg rating of perceived exertion (RPE)	Interval training, Three phases: warm-up, training and cool down; Cycle ergometer	12 weeks	None	30–40 min	90–120 min/week	Yes
Houghton et al., 2016	3 d per week	6–20 point Borg rating of perceived exertion (RPE)	Combination training, Cycling intervals, resistance exercise	12 weeks	None	45–60 min	180 min/week	No
Johnson et al., 2009	3 d per week	50% VO_2_peak for week 1, 60% for week 2, and 70% for weeks 3 and 4.	Continuous training, Cycle ergometer; CG: stretching 3 d per wk.	4 weeks	Intensity	30–45 min	120–180 min/week	Yes
Pugh et al., 2014	3–5 d per week	3 wks 30% HRR; from 4 wk 45% HRR; from 12 wk 60% HRR.	Continuous training, Treadmill, cycle ergometer	16 weeks	Duration, frequency, intensity	30–45 min	90–225 min/week	Progressively yes
Pugh et al., 2013	3–5 d per week	From 1 wk 30% HRR to 60% HRR by 12 wk	Continuous training, Treadmill	16 weeks	Duration, frequency, intensity	30–45 min	90–225 min/week	Progressively yes
Rezende et al., 2016	2 d per week	From VAT up to 10% below RCP	Continuous training, Three phases: warm-up, training and cool down; Treadmill	24 weeks	Duration	40–60 min	80–120 min/week	No
Shamsoddini et al., 2015	3 d per week	From 1 wk 60% HRmax to 75% HRmax by the final wk.	Continuous training, Three phases: warm-up, training and cool down; treadmill	8 weeks	Intensity	45 min	135 min/week	No
Shojaee-Moradie et al., 2016	4–5 d per week	40–60 HRR	Combination training, outdoor aerobic activities, resistance exercise	16 weeks	Duration	20–60 min	80–300 min/week	Progressively yes
Sullivan et al., 2012	5 d per week	45–55% VO_2_peak	Continuous training, Treadmill	16 weeks	Duration	30–60 min	150–300 min/week	Yes
Zhang et al., 2016 (a)	5 d per week	6 mo 65–80% HRmax;	Continuous training, Treadmill	24 weeks	Intensity	30 min	150 min/week	Yes
Zhang et al., 2016 (b)	5 d per week	45–55% HRmax	Continuous training, Treadmill	24 weeks	None	30 min	150 min/week	Yes

* According to American College of Sports Medicine’s guidelines for aerobic exercise testing and prescription (moderate intensity: 5×/wk to total 150–300 min/wk, HR_max_ 64–76% or HRR 40–60% or VO_2max_ 46–63% or RPE 5–6; vigorous intensity: 3×/wk to total 75–150 min/wk, HR_max_ 77–95% or HRR 60–85% or VO_2max_ 63–90% or RPE 7–8) [22].

**Table 4:** Pooled effect sizes based on aerobic exercise intervention in treating MAFLD.

Outcomes	MD	95% CI	*p*-Value	I^2^
ALT (U/L)	−0.87	−2.57, 0.81	0.31	33.9
ALT (U/L) *	−2.4	−4.34, −0.46	0.01	9.1
AST (U/L)	0.02	−1.09, 1.13	0.97	35.7
GGT (U/L)	−0.73	−3.82, 2.36	0.64	0
IHTG (%)	−4.05	−5.37, −2.74	<0.0001	0
HOMA2-IR	−0.06	−0.73, 0.61	0.85	71.7
HOMA-IR	−0.28	−0.88, 0.31	0.35	12.7
BMI (kg/m^2^)	−0.97	−1.40, −0.55	<0.0001	0

* aerobic exercise protocols (continuous, interval) compared to usual care.

## Data Availability

Not applicable.

## Supplementary Materials

The following are available online at https://www.mdpi.com/article/10.3390/jcm10081659/s1, Figure S1: Changes in ALT level, Table S1: Meta-regression analysis result.
