# Supplementary material for: Evidence-Based Aerobic Exercise Training in Metabolic-Associated Fatty Liver Disease: Systematic Review with Meta-Analysis
Source: J Clin Med. 2021 Apr 13;10(8):1659. doi: 10.3390/jcm10081659 (PMC8069623; doi:10.3390/jcm10081659)
Supplement: Supplementary file 1 [file jcm-10-01659-s001.pdf]

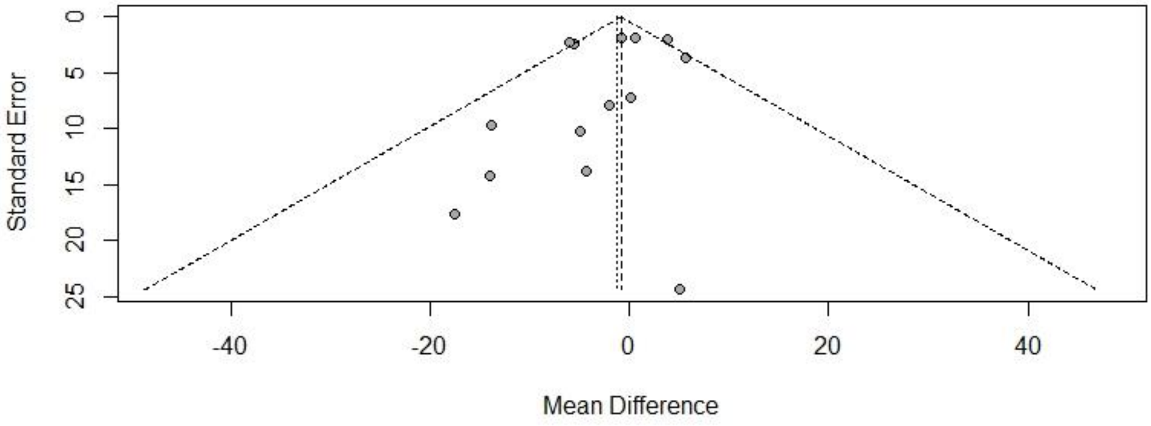

Figure S1. Changes in ALT level (Kendall's tau = -0.1868, p = 0.3880)

Table S1. Meta-regression analysis result

A. ALT Model Results:

factor	estimate	SE	zval	pval	ci.lb	ci.ub
time	5.51	2.73	2.02	0.04	0.15	10.86
intensity	-3.94	17.12	-0.23	0.82	-37.5	29.62
volume	6.55	11.14	0.58	0.56	-15.28	28.38
progression	-4.26	13.90	-0.31	0.76	-31.51	23.00

B. AST Model Results:

factor	estimate	SE	zval	pval	ci.lb	ci.ub
time	4.98	5.28	0.94	0.34	-5.36	15.33
intensity	-7.86	12.67	-0.62	0.53	-32.71	16.98
progression	4.40	12.54	0.36	0.73	-20.20	28.98

C. GGT Model Results

factor	estimate	SE	zval	pval	ci.lb	ci.ub
intensity	-5.31	27.00	-0.20	0.84	-58.25	47.63
progression	2.65	26.57	0.10	0.92	-49.43	54.74

D. BMI Model Results:

factor	estimate	SE	zval	pval	ci.lb	ci.ub
time	2.4	3.07	0.78	0.43	-3.63	8.43
intensity	2.80	3.17	0.88	0.38	-3.42	9.02
volume	-3.47	3.82	-0.91	0.36	-10.96	4.02

E. IHTG Model Results:

factor	estimate	SE	zval	pval	ci.lb	ci.ub
time	-2.25	2.28	-0.98	0.32	-6.73	2.23
